# Supplementary material for: The Use of Autologous Chondrocyte and Mesenchymal Stem Cell Implants for the Treatment of Focal Chondral Defects in Human Knee Joints—A Systematic Review and Meta-Analysis
Source: Int J Mol Sci. 2022 Apr 6;23(7):4065. doi: 10.3390/ijms23074065 (PMC8999850; doi:10.3390/ijms23074065)
Supplement: Supplementary file 1 [file ijms-23-04065-s001.zip › Supplementary Figure S1.pdf]

Supplementary Figure S1. Funnel plot for publication bias

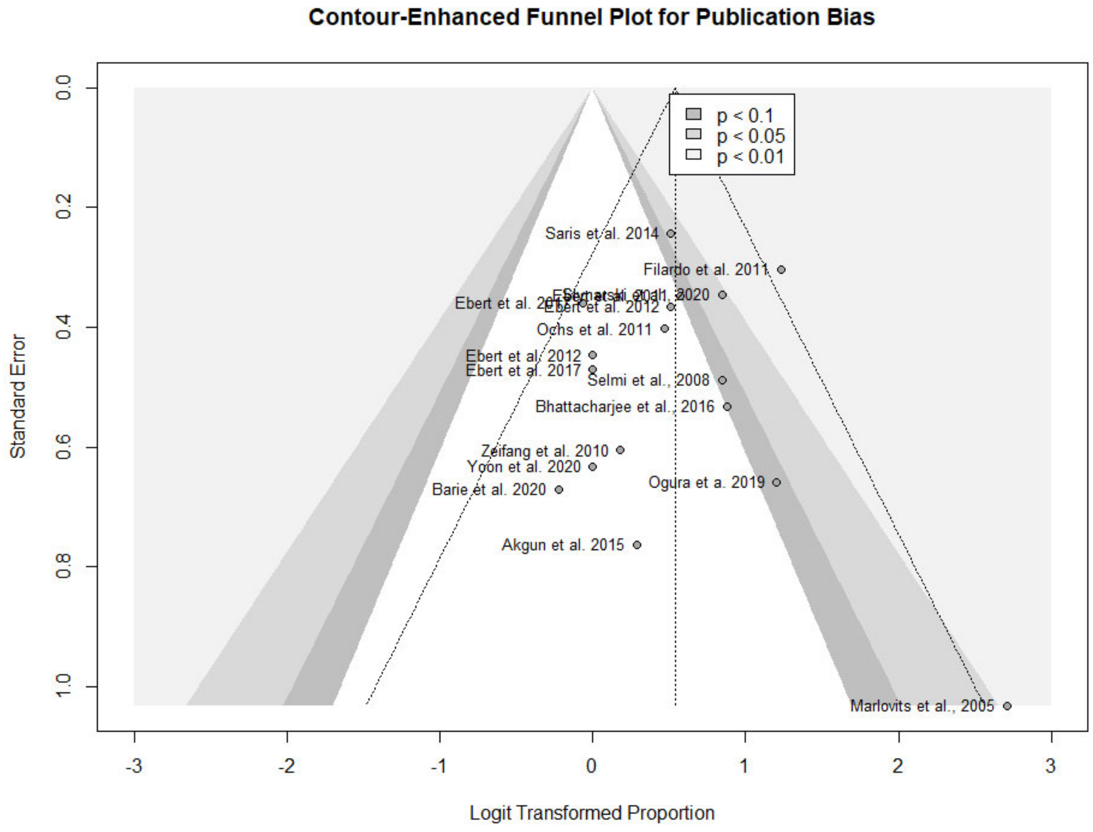

Egger's test of the intercept for publication bias: $p = 0.96$.

Egger's test does not indicate the presence of funnel plot asymmetry.
