# Supplementary material for: The Use of Autologous Chondrocyte and Mesenchymal Stem Cell Implants for the Treatment of Focal Chondral Defects in Human Knee Joints—A Systematic Review and Meta-Analysis
Source: Int J Mol Sci. 2022 Apr 6;23(7):4065. doi: 10.3390/ijms23074065 (PMC8999850; doi:10.3390/ijms23074065)
Supplement: Supplementary file 1 [file ijms-23-04065-s001.zip › Supplementary Table S1.pdf]

Supplementary Table S1: Detailed search strategy

Knee	Cartilage variations	Cartilage to implant variations	Defect variations	MSC or ACI variations	Implant variations	Investigation variations	
1) Knee	2) Chondral<br>3) Cartilage<br>4) Articular<br>5) 2 OR 3 OR 4	6) Cartilage-cartilage<br>7) Cartilage to cartilage<br>8) Cartilage-implant<br>9) Cartilage to implant<br>10) 6 OR 7 OR 8 OR 9	11) Disease<br>12) Defect<br>13) Damage<br>14) Lesion<br>15) 11 OR 12 OR 13 OR 14	16) Mesenchymal stem cell<br>17) MSC<br>18) Bone marrow-derived or bone marrow derived or BM-derived or BM derived<br>19) Adipose-derived or adipose derived or ASC<br>20) Blood-derived or blood derived or peripheral blood-derived or PBSC or PBMSC<br>21) Autologous chondrocyte implantation<br>22) ACI<br>23) P-ACI or periosteum covered ACI or first-generation ACI<br>24) C-ACI or collagen membrane ACI or second-generation ACI<br>25) M-ACI or type I and III collagen membrane ACI or membrane ACI or third generation ACI<br>26) 16 OR 17 OR 18 OR 19 OR 20 OR 21 OR 22 OR 23 OR 24 OR 25	27) Implant<br>28) Scaffold<br>29) Graft<br>30) Inject<br>31) 27 OR 28 OR 29 OR 30	32) Interface<br>33) Integration<br>34) Histology<br>35) Arthroscopy<br>36) MRI or magnetic resonance imaging<br>37) 32 OR 33 OR 34 OR 35 OR 36	38) 1 AND 5 AND 10 AND 15 AND 26 AND 31 AND 37
