# Supplementary material for: The Use of Autologous Chondrocyte and Mesenchymal Stem Cell Implants for the Treatment of Focal Chondral Defects in Human Knee Joints—A Systematic Review and Meta-Analysis
Source: Int J Mol Sci. 2022 Apr 6;23(7):4065. doi: 10.3390/ijms23074065 (PMC8999850; doi:10.3390/ijms23074065)
Supplement: Supplementary file 1 [file ijms-23-04065-s001.zip › Supplementary Table S2.pdf]

Supplementary Table S2: Other MRI outcomes

Author	Intervention	Scoring System	Filling of defect	Surface of Implant	Structure of Implant	Subchondral Lamina	Subchondral Bone
Marlovits et al., 2005 [50]	MACI	Evaluation of cartilage interface	N/A	N/A	N/A	N/A	N/A
Selmi et al., 2008 [51]	MACI	Evaluation of cartilage interface	Complete: 8/15 (53.3%), Partial: 6/15 (40.0%), No filling: 1/15 (6.6%), transition zone no longer distinguishable from adjacent normal tissue: 11/15 (73.3%), mean size of defect decreased from 2.7 cm^2 to 0.4cm^2 at final follow-up (matched t-test, $p < 0.001$)	N/A	N/A	N/A	Bone marrow had normal appearance: 9/15 (60.0 %), bone-marrow oedema: 6/15 (40.0%), subchondral cysts observed: 3/15 (20.0%) patients
Zeifang et al., 2010 [40]	MACI	MOCART	6 months: complete defect filling: 4/8 (50.0%), hypertrophy: 1/8 (12.5%)	N/A	Homogenous at 6 months: 4/8 (50.0%)	Intact subchondral lamina observed: 6 months: 4/8 (50.0%), 12 months: 7/8 (87.5%)	N/A
	ACI-P		6 months: complete defect filling: 1/9 (11.1%), hypertrophy: 7/9 (77.8%)	N/A	Homogenous repair at 12 months: 1/9 (11.1%)	Intact subchondral lamina observed: 6 months: 2/9 (22.2%), 12 months: 5/9 (55.6%)	N/A

Supplementary Table S2: Cont.

Ebert et al., 2011 [38]	MACI & Traditional rehabilitation	MRI Composite Score	3 months: 2.80, 12 months: 3.30, 24 months: 3.30	3 months: 2.81, 12 months: 2.81, 24 months: 2.72,	3 months: 3.53, 12 months: 3.41, 24 months: 3.22	3 months:3.34; 12 months: 3.8; 24 months: 3.97	3 months: 2.75; 12 months: 2.91; 24 months: 2.75
	MACI & Accelerated rehabilitation		3 months: 2.97, 12 months: 3.48, 24 months: 3.40	3 months: 2.90, 12 months: 2.80, 24 months: 2.97	3 months: 3.40, 12 months: 3.80, 24 months: 3.90	3 months: 3.20; 12 months: 3.80; 24 months: 3.90	3 months: 2.97; 12 months: 2.97; 24 months: 2.43
			Time effect p < 0.0001, Group effect p = 0.462, Interaction effect p = 0.878	Time effect p = 0.902, Group effect p = 0.610, Interaction effect p = 0.515	Time effect p = 0.095, Group effect p = 0.861, Interaction effect p = 0.508	Time effect: <0.0001; Group effect: 0.423, Interaction effect: 0.728	Time effect: 0.018, Group effect: 0.937, Interaction effect: 0.096

Supplementary Table S2: Cont.

Ochs et al., 2011 [43]	MACI	MOCART	Filling complete: 8/23 (34.8%), incomplete: 6/23 (26.1%)	Repair tissue surface intact: 8/23 (34.8%), damaged < 50% of depth: 15/23 (65.2%)	Hypertrophy: 9/23 (39.1%), homogenous: 5/23 (21.7%), inhomogeneous: 18/23 (78.2%)	Subchondral lamina irregular or broken: 22/23 (95.7%), completely regular and intact subchondral lamina: 1/23 (4.3%), delamination: 1/23 (4.3%)	Intact: 3/23 (13.0%), Persistent bone marrow edema: 20/23 (87%)
Filardo et al., 2011 [52]	MACI	MOCART	Complete filling: 57.0%	Surface intact: 50.0%	Homogenous: 43.0%	Intact: 45.0%	Subchondral bone changes (oedema, granulation tissue, cysts, and sclerosis): 63.0%
Ebert et al., 2012 [53]	MACI	MRI Composite Score	Rated Good to excellent: 3 months: 12/20 (60.0%), 12 months: 17/20 (85.0%), 24 months: 18/20 (90.0%)<br><br>Rated Poor to fair: 3 months: 8/20 (40.0%), 12 months: 3/20 (15.0%), 24 months: 2/20 (10.0%)	Rated Good to excellent: 3 months: 11/20 (55.0%), 12 months: 13/20 (65.0%), 24 months: 13/20 (65.0%)<br><br>Rated Poor to fair: 3 months: 9/20 (45.0%), 12 months: 7/20 (35.0%), 24 months: 7/20 (35.0%)	Rated Good to excellent: 3 months: 13/20 (65.0%), 12 months: 15/20 (75.0%), 24 months: 15/20 (75.0%)<br><br>Rated Poor to fair: 3 months: 7/20 (35.0%), 12 months: 5/20 (25.0%), 24 months: 5/20 (25.0%)	Rated Good to excellent: 3 months: 17/20 (85.0%), 12 months: 13/20 (65.0%), 24 months: 11/20 (55.0%)<br><br>Rated Poor to fair: 3 months: 3/20 (15.0%), 12 months: 0/20 (0.0%), 24 months: 0/20 (0.0%)	Rated Good to excellent: 3 months: 14/20 (70.0%), 12 months: 13/20 (65.0%), 24 months: 11/20 (55.0%)<br><br>Rated Poor to fair: 3 months: 6/20 (30.0%), 12 months: 7/20 (35.0%), 24 months: 9/20 (45.0%)

Supplementary Table S2: Cont.

Ebert et al., 2012 [39]	MACI & Traditional rehabilitation	MRI Composite Score	Mean: 3.15 (SE: 0.24)	Mean: 2.45 (SE: 0.23)	Mean: 3.15 (SE: 0.22)	Mean: 3.80 (SE: 0.09)	Mean Subchondral bone: 2.65 (SE: 0.25)
	MACI & Accelerated rehabilitation		Mean: 3.23 (SE: 0.17)	Mean: 2.88 (SE: 0.18)	Mean: 3.08 (SE: 0.19)	Mean: 3.75 (SE: 0.09)	Mean Subchondral bone: 2.54 (SE: 0.219)
			Traditional vs. accelerated p = 0.783	Traditional vs. accelerated p = 0.156	Traditional vs. accelerated p = 0.819	Traditional vs. accelerated p = 0.702	Traditional vs. accelerated p = 0.730
Saris et al., 2014 [47]	MACI	N/A	N/A	N/A	N/A	N/A	N/A
	Microfracture		N/A	N/A	N/A	N/A	N/A
Akgun et al., 2015 [42]	Synovium-derived MSCs, CD105+, CD73+, CD90+	MOCART	3 months: 2.93 ± 0.45, 12 months: 3.57 ± 0.35, 24 months: 3.86 ± 0.24. Time effect: p = 0.002	3 months: 3.43 ± 0.53, 12 months: 3.14 ± 0.38, 24 months: 3.71 ± 0.49. Time effect: 0 = 0.05	:3 months: 3.29 ± 0.49, 12 months: 3.14 ± 0.38, 24 months: 3.36 ± 0.48. Time effect: p = 0.549	3 months: 2.86 ± 0.38, 12 months: 3.14 ± 0.38, 24 months: 3.43 ± 0.53. Time effect: p = 0.146	3 months: 2.57 ± 0.53, 12 months: 3.00 ± 0.58, 24 months: 3.29 ± 0.76. Time effect: p = 0.037
	Cartilage-derived chondrocytes, CD44+, CD73+		3 months: 2.64 ± 0.63, 12 months: 3.07 ± 0.19, 24 months: 3.29 ± 0.27. Time effect: p = 0.162	3 months: 3.00 ± 0.00, 12 months: 2.57 ± 0.53, 24 months: 2.57 ± 0.53. Time effect: p = 0.549	3 months: 3.00 ± 0.00, 12 months: 2.86 ± 0.38, 24 months: 3.00 ± 0.00. Time effect: p = 0.368	3 months: 2.29 ± 0.49, 12 months: 3.21 ± 0.27, 24 months: 3.29 ± 0.49. Time effect: p = 0.006	3 months: 2.43 ± 0.53, 12 months: 2.57 ± 0.53, 24 months: 2.71 ± 0.49. Time effect: p = 0.223

Supplementary Table S2: Cont.

			Group effect: 3 months: p = 0.389, 12 months: p = 0.10, 24 months: p =0.005	Group effect: 3 months: p = 0.6, 12 months: p = 0.044, 24 months: p = 0.005	Group effect: 3 months: p = 0.141, 12 months: p = 0.173, 24 months: p = 0.061	Group effect: 3 months: p = 0.037, 12 months: p = 0.375, 24 months: p = 0.591	Group effect: 3 months: p = 0.606, 12 months: p = 0.174, 24 months: p = 0.116
Bhattacharjee et al., 2016 [44]	MACI	MOCART	Complete: 4/11 (44.4%), hypertrophy: 1/11 (11.1%), <50% of adjacent cartilage: 2/11 (22.2%), >50% of adjacent cartilage: 2/11 (22.2%), subchondral bone exposed: 2/11 (22.2%)	Intact: 0/11 (0.0%), damaged <50% of depth: 7/11 (77.8%), damaged >50% of depth: 4/11 (44.4%)	Homogeneous: 4/11 (36.4%), Inhomogeneous (0/5): 7/11 (63.6%)	Intact: 3/11 (27.3%), Partially formed: 8/11 (72.7%)	Intact: 1/11 (9.1%), Not intact: 10/11 (90.1%)
Ebert et al., 2017 [49]	MACI	MOCART	3 months: 2.85 ± 0.15, 1 years: 3.34 ± 0.14, 2 years: 3.39 ± 0.14, 5 years: 3.39 ± 0.16, time effect: p = 0.33	3 months: 2.90 ± 0.20, 1 years: 2.84 ± 0.22, 2 years: 2.97 ± 0.21, 5 years: 2.87 ± 0.21, time effect: = 0.975	3 months: 3.06 ± 0.20, 1 years: 3.23 ± 0.16, 2 years: 3.13 ± 0.18, 5 years: 3.16 ± 0.19, time effect: p = 0.939	3 months: 3.00 ± 0.10, 1 years: 3.71 ± 0.10, 2 years: 3.77 ± 0.08, 5 years: 3.65 ± 0.09, time effect: p = <0.0001	3 months: 2.77 ± 0.14, 1 years: 2.65 ± 0.21, 2 years: 2.58 ± 0.22, 5 years: 2.81 ± 0.20, time effect: p = 0.822

Supplementary Table S2: Cont.

Ebert et al., 2017 [54]	ACI and 6-week return to full weight-bearing	MOCART	3 months: 3.09 ± 0.16, 12 months: 3.53 ± 0.18, 24 months: 3.47 ± 0.18	3 months: 3.41 ± 0.23, 12 months: 3.18 ± 0.27, 24 months: 3.65 ± 0.25	Structure of the implant: 3 months: 3.59 ± 0.21, 12 months: 3.24 ± 0.23, 24 months: 3.58 ± 0.23	3 months: 3.06 ± 0.13, 12 months: 3.47 ± 0.16, 24 months: 3.59 ± 0.14	3 months: 0.294 ± 0.18, 12 months: 3.41 ± 0.20, 24 months: 3.35 ± 0.22
	ACI and 8-week return to full weight-bearing		3 months: 2.71 ± 0.16, 12 months: 3.18 ± 0.17, 24 months: 3.18 ± 0.17	3 months: 2.95 ± 0.22, 12 months: 2.63 ± 0.26, 24 months: 2.79 ± 0.24	Structure of the implant: 3 months: 3.26 ± 0.20, 12 months: 3.16 ± 0.21, 24 months: 3.16 ± 0.21.	3 months: 2.84 ± 0.12, 12 months: 3.31 ± 0.15, 24 months: 3.42 ± 0.13	3 months: 2.79 ± 0.17, 12 months: 3.00 ± 0.19, 24 months: 3.05 ± 0.20
			Time effect: p = 0.002, Group effect: p = 0.117, Interaction effect: p = 0.717	Time effect: p = 0.846, Group effect: p = 0.022, Interaction effect: p = 0.327	Time effect: p = 0.720, Group effect: p = 0.192, Interaction effect: p = 0.720	Time effect: p < 0.0001, Group effect: p = 0.227, Interaction effect: p = 0.834	Time effect: p = 0.038, Group effect: p = 0.208, Interaction effect: p = 0.638
Ogura et al., 2019 [45]	ACI segmental sandwich technique	MOCART	Complete: 7/10 (70.0%), <50% of adjacent cartilage: 3/10 (30.0%)	Intact: 8/10 (80.0%), damaged <50% of depth: 1/10 (10.0%), damaged >50% of depth: 1/10 (10.0%)	Structure of the implant: Homogeneous: 4/11 (36.4%), inhomogeneous (0/5): 7/11 (63.6%)	5/5: 4/10 (40.0%), 0/5: 6/10 (60.0%)	5/5: 6/10 (60.0%), 0/5 - 4/10 (40.0%)

Supplementary Table S2: Cont.

Yoon et al., 2020 [46]	GACI	MOCART	3 months: complete: 2/10 (20.0%), hypertrophy: 0/10 (10.0%), >50.0% of adjacent cartilage: 4/10 (40.0%), <50.0% of adjacent cartilage: 2/10 (20.0%), subchondral bone exposed: 2/10 (20.0%)	3 months: intact: 5/10 (50.0%), damaged <50.0% of repair tissue depth: 2/10 (20.0%), >50.0% of depth or total degeneration: 3/10 (30.0%)	3 months: homogenous: 4/10 (40.0%), inhomogeneous or cleft formation: 6/10 (60.0%)	3 months: intact: 6/10 (60.0%), non-intact: 4/10 (40.0%)	3 months: intact: 5/10 (50.0%), non-intact: 5/10 (50.0%)
			6 months: complete: 3/10 (30.0%), > 50.0% of adjacent cartilage: 5/10 (50.0%), Subchondral bone exposed: 2/10 (20.0%)	6 months: intact: 5/10 (50.0%), damaged <50.0% of repair tissue depth: 2/10 (20.0%), >50.0% of depth or total degeneration: 3/10 (30.0%)	6 months: homogenous: 4/10 (40.0%), inhomogeneous or cleft formation: 6/10 (60.0%)	6 months: intact: 6/10 (60.0%), non-intact: 4/10 (40.0%)	6 months: intact: 5/10 (50.0%), non-intact: 5/10 (50.0%)
			12 months: complete: 7/10 (70.0%), >50.0% of adjacent cartilage: 2/10 (20.0%), Subchondral bone exposed: 1/10 (10.0%)	12 months: intact: 7/10 (70.0%), damaged <50.0% of repair tissue depth: 3/10 (30.0%)	12 months: homogenous: 8/10 (80.0%), inhomogeneous or cleft formation: 2/10 (20.0%)	12 months: intact: 9/10 (90.0%), non-intact: 1/10 (10.0%)	12 months: intact: 7/10 (70.0%), non-intact: 3/10 (30.0%)
			24 months: complete: 10/10 (100.0%)	24 months: intact: 8/10 (80.0%), damaged <50.0% of repair tissue depth: 2/10 (20.0%)	24 months: homogenous: 8/10 (80.0%), inhomogeneous or cleft formation: 2/10 (20.0%)	24 months: intact: 9/10 (90.0%), non-intact: 1/10 (10.0%)	24 months: intact: 8/10 (80.0%), non-intact: 2/10 (20.0%)

Supplementary Table S2: Cont.

Słynarski et al., 2020 [48]	ACI combined with bone marrow mononucleated cells	MOCART	3 months: mean percentage of lesion fill: 98.3% ± 10.2%	3 months: observer 1: intact: 18/38 (47.4%), damaged <50% of depth: 16/38 (42.1%), >50% of depth: 4/38 (10.5%); observer 2: 28/40 (70.0%), 12/40 (30%), 0/40 (0.0%)	3 months: observer 1: homogeneous: 25/38 (65.8%), inhomogeneous: 13/38 (34.2%); observer 2: 18/40 (45%), 22/40 (55%)	3 months: observer 1: intact: 0/39 (0.0%), not intact 39/39 (100.0%). observer 2: 0/40 (0.0%), 40/40 (100.0%)	3 months: observer 1: intact: 2/39 (5.1%), edema, gt, cysts, sclerosis: 37/39 (94.8%). observer 2: 3/40 (7.5%), 37/40 (92.5%)
Słynarski et al., 2020 [48]	ACI combined with bone marrow mononucleated cells	MOCART	6 months: mean percentage of lesion fill: 98.9% ± 13.1%	6 months: observer 1: 14/38 (36.8%), 21/38 (55.3%), 3/38 (7.9%). observer 2: 28/38 (73.7%), 10/38 (26.3%), 0/38 (0.0%)	6 months: observer 1: 20/38 (52.6%), 18/38 (47.4%). observer 2: 25/38 (65.8%), 13/38 (34.2%)	6 months: observer 1: 0/38 (0.0%), 38/38 (100.0%). observer 2: 0/38 (0.0%), 38/38 (100.0%)	6 months: observer 1: 12/38 (31.6%), 26/38 (68.4%). observer 2: 9/38 (23.7%), 29/38 (76.3%)
Słynarski et al., 2020 [48]	ACI combined with bone marrow mononucleated cells	MOCART	12 months: mean percentage of lesion fill: 101% ± 6.6%	12 months: observer 1: 18/37 (48.6%), 18/37 (48.6%), 1/37 (2.7%). observer 2: 30/37 (81.1%), 7/37 (18.9%), 0/37 (0.0%)	12 months: observer 1: 11/37 (29.7%), 26/37 (70.3%); observer 2: 22/36 (61.1%), 14/36 (38.9%)	12 months: observer 1: 0/37 (0.0%), 37/37 (100.0%). observer 2: 0/36 (0.0%), 36/36 (100.0%)	12 months: observer 1: 16/37 (43.2%), 21/37 (56.8%). observer 2: 12/36 (33.3%), 24/36 (66.7%)
Słynarski et al., 2020 [48]	ACI combined with bone marrow mononucleated cells	MOCART	24 months: mean percentage of lesion fill: 96.9% ± 11.9%	24 months: observer 1: 15/32 (46.9%), 15/32 (46.9%), 2/32 (6.3%). observer 2: 24/31 (77.4%), 6/31 (19.4%), 1/31 (3.2%)	24 months: observer 1: 7/32 (21.9%), 25/32 (78.1%). observer 2: 19/31 (61.3%), 12/31 (38.7%)	24 months: observer 1: 0/32 (0%), 32/32 (100.0%). observer 2: 0/31 (0%), 31/31 (100.0%)	24 months: observer 1: 23/32 (71.9%), 9/32 (28.1%). observer 2: 14/31 (45.2%), 17/31 (54.8%)

Supplementary Table S2: Cont.

Barié et al., 2020 [41]	MACI	MOCART	Complete: 5/9 (55.5%), incomplete < 50% of the adjacent cartilage: 1/9 (11.1%), subchondral bone exposed: 3/9 (33.4%)	Surface intact: 2/9 (22.2%), surface damaged <50% of repair tissue depth: 3/9 (33.4%), surface damaged >50% of repair tissue depth: 4/9 (44.4%	Homogeneous: 3/9 (33.4%), inhomogeneous or cleft formation: 6/9 (66.6%)	Intact: 8/9 (88.9%), not intact: 1/9 (11.1%)	Intact: 1.9 (11.1%), not intact: 8/9 (88.9%)
	ACI-P		Complete: 5/7 (71.4%), hypertrophy: 1/7 (14.3%), subchondral bone exposed: 1/7 (14.3%)	Surface intact: 3/7 (42.8%), surface damaged <50% of repair tissue depth: 3/7 (42.8%), surface damaged >50% of repair tissue depth: 1/7 (14.3%)	Homogeneous: 3/7 (42.8%), inhomogeneous or cleft formation: 4/7 (57.2%)	Intact: 6/7 (85.7%), not intact: 1/7 (14.3%)	Intact: 0/7 (0.0%), not intact: 7/7 (100.0%)

Mean values are used unless specified. Abbreviations: SE, standard error.
