# Supplementary material for: The Use of Autologous Chondrocyte and Mesenchymal Stem Cell Implants for the Treatment of Focal Chondral Defects in Human Knee Joints—A Systematic Review and Meta-Analysis
Source: Int J Mol Sci. 2022 Apr 6;23(7):4065. doi: 10.3390/ijms23074065 (PMC8999850; doi:10.3390/ijms23074065)
Supplement: Supplementary file 1 [file ijms-23-04065-s001.zip › Supplementary Table S3.pdf]

Supplementary Table S3: Clinical outcomes

Author	Intervention	IKDC	KOOS (Pain)	KOOS (Symptoms)	Lysholm	TAS	VAS (Total or Severity)	SF-36 Physical Health	SF-36 Mental Health	Complications
Marlovits et al., 2005 [50]	MACI	N/A	N/A	N/A	N/A	N/A	N/A	N/A	N/A	N/A
Selmi et al., 2008 [51]	MACI	Preoperative mean: 37.0 (range 18.0 to 64.0), follow-up mean: 77.8 (range 42.0 to 96.0), matched t-test p < 0.001;	N/A	N/A	N/A	N/A	N/A	N/A	N/A	N/A
Zeifang et al., 2010 [40]	MACI	Post-operative change 12 months: 20.9 ± 20.9, m-ACI vs ACI-P Wilcoxon-Mann-Whitney Test, p= 0.5573<br><br>Post-operative change 24 months: 19.0 ± 26.8, m-ACI vs ACI-P Wilcoxon-Mann-Whitney Test, p= 0.4994	N/A	N/A	Post-operative change 12 months: 4.9 ± 19.0, m-ACI vs ACI-P Wilcoxon-Mann-Whitney Test, p= 0.0449<br><br>Post-operative change 24 months: 1.2 ± 22.3, m-ACI vs ACI-P Wilcoxon-Mann-Whitney Test, p= 0.0487	Post-operative change 12 months: 0.1 ± 2.1, m-ACI vs ACI-P Wilcoxon-Mann-Whitney Test, p= 0.4063<br><br>Post-operative change 12 months: 0.6 ± 2.7, m-ACI vs ACI-P Wilcoxon-Mann-Whitney Test, p= 0.1043	N/A	N/A	N/A	Effusion observed at 6 months: 5/11 (45.5%), transplant hypertrophy at 6 months: 1/11 (9.1%), transplant hypertrophy at 12 months 6/11 (54.5%), revision arthroscopy: 3/11 (27.3%)

Supplementary Table S3: Cont.

	ACI-P	Post-operative change 12 months: 24.6 ± 19.3, m-ACI vs ACI-P Wilcoxon-Mann-Whitney Test, p= 0.5573<br>Post-operative change 24 months: 25.2 ± 23.2, m-ACI vs ACI-P Wilcoxon-Mann-Whitney Test, p= 0.4994	N/A	N/A	Post-operative change 12 months: 25.0 ± 22.8, m-ACI vs ACI-P Wilcoxon-Mann-Whitney Test, p= 0.0449<br>Post-operative change 24 months: 22.7 ± 25.9, m-ACI vs ACI-P Wilcoxon-Mann-Whitney Test, p= 0.0487	Post-operative change 12 months: 0.9 ± 2.5, m-ACI vs ACI-P Wilcoxon-Mann-Whitney Test, p= 0.4063<br>Post-operative change 12 months: 1.7 ± 2.0, m-ACI vs ACI-P Wilcoxon-Mann-Whitney Test, p= 0.1043	N/A	N/A	N/A	Delamination observed by MRI at 6 months 1/9 (11.1%), effusion observed at 6 months: 6/9 (66.7%), transplant hypertrophy at 6 months in 7/9 (77.8%), transplant hypertrophy at 12 months 6/9 (66.7%)
Ebert et al., 2011 [38]	MACI & Traditional rehabilitation	N/A	24 months post-operation: 82.5, traditional vs. accelerated p= 0.422	24 months post-operation: 82.86, traditional vs. accelerated p= 0.197	N/A	N/A	N/A	24 months post-operation: 47.02, traditional vs. accelerated p= 0.289	24 months post-operation: 56.11, traditional vs. accelerated p= 0.669	Graft hypertrophy: 3 months: 1/34 (2.9%), 12 months: 5/34 (14.7%), 24 months: 11/34 (32.4%); 24 months demonstrating subchondral bed devoid of any significant repair tissue: 1/34 (2.9%)

Supplementary Table S3: Cont.

	MACI & Accelerated rehabilitation	N/A	24 months post-operation: 86.15, traditional vs. accelerated p= 0.422	24 months post-operation: 8.04, traditional vs. accelerated p= 0.197	N/A	N/A	N/A	24 months post-operation: 49.79, traditional vs. accelerated p= 0.289	24 months post-operation: 55.95, traditional vs. accelerated p= 0.669	Graft hypertrophy: 3 months: 2/35 (5.7%), 12 months: 6/35 (17.1%), 24 months: 8/35 (22.9%); graft loss between 6 and 9 months: 1/35 (2.9%), 24 months post-surgery subchondral bed devoid of any significant repair tissue: 1/35 (2.9%)
Ochs et al., 2011 [43]	MACI	IKDC subjective score: pre-operatively: 50.5% ± 16.1, latest follow-up 78.4 ± 13.4%; IKDC objective score: normal: 8/26 (30.8%), nearly normal: 15/26 (57.7%), abnormal: 2/26 (7.7%), severely abnormal: 1/26 (3/8%)	N/A	N/A	Pre-operatively: 53.2 ± 18.0, Time of follow-up: 88.5 ± 9.5, p < 0.001	Pre-operatively: 3.5 ± 0.8, Time of follow-up: 4.6 ± 1.2, p= 0.002	N/A	N/A	N/A	No complications noted

Supplementary Table S3: Cont.

Filardo et al., 2011 [52]	MACI	IKDC Subjective score: Basal level: 39.6 ± 15.0, 12 months: 73.6 ± 18.8, 24 months: 76.5 ± 20.7, 84 months: 77.3 ±21.5<br><br>IKDC objective score before treatment level: 21% normal and nearly normal knees, 12 months: 90%, 84 months: 93%, p < 0.0005	N/A	N/A	N/A	Tegner sport Activity level pre-treatment: 1.7 ± 1.3, 84-months: 5.7 ± 2.6, pre-injury level: 6.9 ±2.0, p < 0.0005	Basal evaluation: 61.2 ± 16.7, 12 months: 81.9 ± 16.6, p < 0.0005, 84 months: 84.3 ± 15.3	N/A	N/A	Failed grafts: 7/62 (11.3%), adhesions: 3/62 (4.8%)
Ebert et al., 2012 [53]	MACI	N/A	Before surgery: 58.06 (SE: 5.57), 3 months: 76.17 (SE: 3.07), 6 months: 80.92 (SE: 2.75), 12 months: 80.07 (SE: 3.24), 24 months: 86.81 (SE: 2.04), p < 0.0001	Before surgery: 59.46 (SE: 4.38), 3 months: 83.93 (SE: 2.57), 6 months: 87.5 (SE: 1.94), 12 months: 85.3 (SE: 2.15), 24 months: 85.94 (SE: 2.43), p < 0.0001	N/A	N/A	Before surgery: 5.48 (SE: 0.55), 3 months: 3.26 (SE: 0.48), 6 months: 2.24 (SE: 0.48), 12 months: 2.21 (SE: 0.35), 24 months: 1.94 (SE: 0.28), p < 0.0001	Before surgery: 38.51 (SE: 2.52), 3 months: 39.43 (SE: 2.81), 6 months: 43.84 (SE: 1.86), 12 months: 47.21 (SE: 1.87), 24 months: 49.98 (SE: 1.64), p= 0.001	Before surgery: 49.79 (SE: 1.79), 3 months: 53.2 (SE: 2.17), 6 months: 55.47 (SE: 1.67), 12 months: 53.66 (SE: 1.89), 24 months: 55.19 (SE: 1.09), p= 0.194	Graft hypertrophy: 3 months: 5%, 12 months: 20%, 24 months: 20% (all medial femoral), graft failure on lateral condyle: 1/20 (5.0%)

Supplementary Table S3: Cont.

Ebert et al., 2012 [39]	MACI & Traditional rehabilitation	N/A	86.04 (SE: 2.34), traditional vs. accelerated p= 0.892	83.50 (SE: 4.23), traditional vs. accelerated p= 0.545	N/A	N/A	2.29 (SE: 0.31), traditional vs. accelerated rehabilitation p= 0.905	47.74 (SE: 2.02), traditional vs. accelerated rehabilitation p= 0.665	54.00 (SE: 1.64), traditional vs. accelerated rehabilitation p= 0.506	Graft failures: 3/29 (10.3%)
	MACI & Accelerated rehabilitation	N/A	85.49 (SE: 3.06), traditional vs. accelerated p= 0.545	86.36 (SE: 13.07), traditional vs. accelerated p= 0.545	N/A	N/A	2.22 (SE: 0.40), traditional vs. accelerated rehabilitation p= 0.905	48.87 (SE: 1.66), traditional vs. accelerated rehabilitation p= 0.665	55.42 (SE: 1.36), traditional vs. accelerated rehabilitation p= 0.506	Graft failures: 2/29 (6.9%)
Saris et al., 2014 [47]	MACI	Baseline (n = 71): 32.9 ± 13.3, year 2 (n = 72): 65.7 ± 18.5, MACI vs MFX Improvement p= 0.069	Baseline (n = 72): 37.0 ± 13.5 (n:72), year 2 (n = 72): 82.5 ± 16.2, MACI vs MFX Improvement p < 0.001	Baseline (n = 72): 14.9 ± 14.7 (n:72), year 2 (n = 72): 60.9 ± 27.8, MACI vs MFX Improvement p < 0.001	N/A	N/A	N/A	SF-12: Baseline (n = 72): -1.77 ± 0.86, year 2 (n = 72): -0.32± 0.89, MACI vs MFX Improvement p= 0.001	SF-12: Baseline (n = 72): 0.04 ± 1.2, year 2 (n = 72): 0.45± 0.9, MACI vs MFX Improvement p= 0.523	Not Reported
	Microfracture	Baseline (n = 72): 29.3 ± 13.4, year 2 (n = 71): 58.8 ± 22.3, MACI vs MFX Improvement p= 0.069	Baseline (n = 71): 35.5 ± 12.1, year 2 (n = 70): 70.9 ± 24.2, MACI vs MFX Improvement p < 0.001	Function: Baseline (n = 71): 12.6 ± 16.7, year 2 (n = 70): 48.7 ± 30.3, MACI vs MFX Improvement p < 0.001	N/A	N/A	N/A	SF-12: Baseline (n = 69): -1.93 ± 0.82, year 2 (n = 71): -0.82± 1.12, MACI vs MFX Improvement p= 0.001	SF-12: Baseline (n = 69): -0.17 ± 1.3, year 2 (n = 71): 0.49± 1.0, MACI vs MFX Improvement p= 0.523	
Akgun et al., 2015 [42]	Synovium-derived MSCs, CD105+, CD73+, CD90+	N/A	Pre-operative: 63.49 ± 2.50, 6 months: 81.35 ± 3.09, 12 months: 86.51 ± 3.37, 24 months: 88.10 ± 2.64. Time effect: P= 0	Pre-operative: 66.33 ± 3.49, 6 months: 77.55 ± 5.34, 12 months: 87.24 ± 4.05, 24 months: 89.80 ± 4.34. Time effect: P= 0	N/A	N/A	Severity: Pre-operative: 4.86 ± 0.69, 6 months: 2.00 ± 0.58, 12 months: 1.29 ± 0.49, 24 months: 0.57 ± 0.53. Time effect. P= 0	N/A	N/A	Not Reported

Supplementary Table S3: Cont.

	Cartilage-derived chondrocytes, CD44+, CD73+	N/A	Pre-operative: 67.46 ± 2.64, 6 months: 66.27 ± 3.74, 12 months: 81.75 ± 2.71, 24 months: 82.54 ± 3.48. Time effect: P= 0 Group effect: p= Pre-operative: p= 0.021, 6 months: p= 0.002, 12 months: p= 0.018, 24-months: p= 0.009	Pre-operative: 67.46 ± 2.64, 6 months: 61.22 ± 6.33, 12 months: 81.63 ± 3.21, 24 months: 83.67 ± 2.81. Time effect: P= 0 Group effect: p= Pre-operative: p= 0.558, 6 months: p= 0.002, 12 months: p= 0.020, 24 months: p= 0.015	N/A	N/A	Severity: Pre-operative: 4.71 ± 1.11, 6 months: 3.43 ± 1.13, 12 months: 1.57 ± 0.53, 24-months: 1.14 ± 0.69. Time effect: P= 0.001 Group effect: p= Pre-operative: p= 0.839, 6 months: p= 0.02, 12 months: p= 0.298, 24 months: p= 0.114	N/A	N/A	Not Reported
Bhattacharjee et al., 2016 [44]	MACI	N/A	N/A	N/A	Pre-operative: 45 (IQR, 24), 1 year: 77 (IQR, 35), 5 years: 70 (IQR, 35)	N/A	N/A	N/A	N/A	Not Reported
Ebert et al., 2017 [49]	MACI	N/A	Pre-operative: 59.6 ± 3.9, 3 months: 76.1 ± 2.4, 1 years: 84.3 ± 1.9, 2 years: 89.3 ± 1.5, 5 years: 91.2 ± 1.8, time effect: p < 0.0001	Pre-operative: 62.3 ± 3.4, 3 months: 80.9 ± 1.9, 1 years: 87.0 ± 1.5, 2 years: 87.2 ± 1.5, 5 years: 85.6 ± 2.1, time effect: p < 0.0001	Pre-operative: 53.8 ± 6.9, 3 months: 65.5 ± 7.5, 1 years: 76.3 ± 4.7, 2 years: 82.3 ± 4.0, 5 years: 86.8 ± 4.2, time effect: p < 0.0001	Pre-operative: 2.7 ± 0.3, 3 months: 2.9 ± 0.4, 1 years: 3.4 ± 0.3, 2 years: 4.5 ± 0.5, 5 years: 5.5 ± 0.5, time effect: t < 0.0001	Severity: Pre-operative: 5.7 ± 0.4, 3 months: 2.8 ± 0.4, 1 years: 2.2 ± 0.3, 2 years: 1.7 ± 0.2, 5 years: 1.7 ± 0.3, time effect: P < 0.0001	N/A	3 months: 40.7 ± 1.9 [Pre-operative: 39.1 ± 1.9] 1 years: 48.6 ± 1.2 2 years: 51.0 ± 1.0 5 years: 51.0 ± 1.4, time effect: p < 0.0001	Hypertrophic graft: 3 months: 3/30 (10.0%), 5 years: 7/30 (23.3%). Graft failure: 2/30 (6.7%)

Supplementary Table S3: Cont.

Ebert et al., 2017 [54]	ACI and 6-week return to full weight-bearing	N/A	Pre-operative: 63.2 ± 3.9, 4 weeks: 70.2 ± 4.6, 8 weeks: 77.9 ± 3.6, 3 months: 80.1 ± 3.4, 6 months: 86.2 ± 2.3, 12 months: 86.8 ± 2.1, 24 months: 88.0 ± 3.3	Pre-operative: 64.0 ± 4.3, 4 weeks: 71.7 ± 4.0, 8 weeks: 77.9 ± 2.9, 3 months: 80.5 ± 2.6, 6 months: 86.2 ± 3.2, 12 months: 85.8 ± 2.9, 24 months: 86.1 ± 2.5	N/A	N/A	Severity: pre-operative: 5.7 ± 0.6, 4 weeks: 2.9 ± 0.6, 8 weeks: 2.3 ± 0.5, 3 months: 1.4 ± 0.4, 6 months: 1.9 ± 0.4, 12 months: 2.0 ± 0.4, 24 months: 1.7 ± 0.4	Pre-operative: 35.1 ± 1.9, 4 weeks: 30.9 ± 1.8, 8 weeks: 39.7 ± 2.1, 3 months: 41.6 ± 2.0, 6 months: 45.6 ± 2.1, 12 months: 46.7 ± 1.8, 24 months: 49.1 ± 2.5	Pre-operative: 51.1 ± 2.4, 4 weeks: 52.7 ± 2.9, 8 weeks: 56.8 ± 1.9, 3 months: 57.5 ± 1.5, 6 months: 57.4 ± 1.5, 12 months: 59.3 ± 1.4, 24 months: 54.9 ± 1.7	Graft hypertrophy: 9/35 evaluated at 2 months (4 in the 6-week group, 5 in the 8-week group), Graft failure: 2 at 24 months in 8-week group
	ACI and 8-week return to full weight-bearing	N/A	Pre-operative: 66.9 ± 4.2, 4 weeks: 65.5 ± 4.9, 8 weeks: 77.3 ± 3.6, 3 months: 75.5 ± 3.6, 6 months: 82.6 ± 2.5, 12 months: 88.3 ± 2.2, 24 months: 90.2 ± 3.6<br>Time effect: p < 0.0001, Group effect: p = 0.865, Interaction effect: p = 0.703	Pre-operative: 73.1 ± 4.6, 4 weeks: 68.4 ± 4.3, 8 weeks: 78.8 ± 3.1, 3 months: 77.5 ± 2.8, 6 months: 82.9 ± 3.4, 12 months: 88.7 ± 3.1, 24 months: 88.8 ± 2.6<br>Time effect: p < 0.001, Group effect: p = 0.778, Interaction effect: p = 0.680	N/A	N/A	Severity: pre-operative: 5.3 ± 0.6, 4 weeks: 4.5 ± 0.6, 8 weeks: 3.4 ± 0.6, 3 months: 3.0 ± 0.4, 6 months: 2.4 ± 0.4, 12 months: 1.8 ± 0.4, 24 months: 1.8 ± 0.4<br>Time effect: p < 0.0001, Group effect: p = 0.702, Interaction effect: p = 0.835	Pre-operative: 39.6 ± 2.1, 4 weeks: 30.6 ± 1.9, 8 weeks: 31.6 ± 2.2, 3 months: 37.2 ± 2.2, 6 months: 44.8 ± 2.3, 12 months: 49.6 ± 1.9, 24 months: 50.2 ± 2.7<br>Time effect: p = 0.004, Group effect: p = 0.839, Interaction effect: p = 0.844	Pre-operative: 51.5 ± 2.6, 4 weeks: 51.8 ± 3.2, 8 weeks: 58.6 ± 2.1, 3 months: 58.8 ± 1.6, 6 months: 56.9 ± 1.6, 12 months: 57.1 ± 1.5, 24 months: 58.0 ± 1.9<br>Time effect: p <.0001, Group effect: p = 0.193, Interaction effect: p = 0.472	

Supplementary Table S3: Cont.

Ogura et al., 2019 [45]	ACI segmental sandwich technique	N/A	N/A	N/A	N/A	N/A	Pre-operative: 7.3 ± 1.9, Final Follow-up: 2.8 ± 1.5, p < 0.001 (includes patellar lesions)	Pre-operative: 36.3 ± 8.9, Final Follow-up: 47.8 ± 8.6, p < 0.001 (includes patellar lesions)	Pre-operative: 47.3 ± 7.9, Final Follow-up: 53.8 ± 6.6, p = .0116 (includes patellar lesions)	No complications reported
Yoon et al., 2020 [46]	GACI	Pre-operative: 46.6 ± 18.1, 5 years: 63.7 ± 15.2, time effect: p = 0.041	Pre-operative: 64.7 ± 16.0, 3 months: 75, 6 months: 68, 12 months: 77, 24 months: 81, 5 years: 79.2 ± 7.3, time effect: p = 0.025	Pre-operative: 56.8 ± 14.2, 3 months: 58, 6 months: 55, 12 months: 60, 24 months: 67, 5 years: 71.8 ± 17.7, time effect: not statistically significant	N/A	N/A	Pre-operative: 38.0 ± 18.6, 5 years: 20.5 ± 10.1, time effect: p = 0.045	N/A	N/A	No complications reported
Słynarski et al., 2020 [48]	ACI combined with bone marrow mononucleated cells	Pre-operative: 45 (IQR: 30 – 53), 3 months: 44 (IQR: 37 – 51), 6 months: 52 (IQR: 42 – 61), 12 months: 58 (IQR: 45 – 71), 24 months: 70 (IQR: 53 – 83)	Pre-operative: 62, 3 months: 67, 6 months: 74, 12 months: 75, 24 months: 80	Pre-operative: 62, 3 months: 65, 6 months: 69, 12 months: 73, 24 months: 81	N/A	N/A	Pre-operative: 52 (IQRL 34 – 60), 3 months (IQR: 15 – 50), 6 months: 21 (IQR: 7 – 42), 12 months: 18 (IQR: 6 – 37), 24 months: 15 (IQR: 4 – 38)	N/A	N/A	Arthralgia: 12/40 (30%), Joint effusion: 7/40 (17.5%), Joint swelling: 5/40 (12.5%), Decreased ROM: 2/40 (5%), Arthrofibrosis: 2/40 (5%), Delamination of scaffold: 1/40 (2.5%), Post-operative adhesions: 1/40 (2.5%)

Supplementary Table S3: Cont.

	MACI	Pre-operative: 46.1 ± 2.5, 12 months: 69.1 ± 23.6, 24 months: 65.1 ± 29.4, last follow-up: 70.4 ± 19.3. Time effect: p = 0.021	N/A	N/A	Pre-operative: 65.1± 21.6, 12 months: 88±29, 24 months: 78±29, last follow-up: 72.4±16.4. Time effect: p = 0.374	Pre-operative: 4.6 ± 2.7, 12 months: 3.9 ± 2, 24 months: 4.2 ± 3, last follow-up: 4.9 ± 2.3. Time effect: p = 0.498	Pre-operative: 29.2±1.3, 12 months: 30.9±1.8, 24 months: 31±2.3, last follow-up: 31.5±1.2. Time effect: p = 0.008	Pre-operative: 12.1±0.5, 12 months: 11.8±0.3, 24 months: 11.6±0.7, last follow-up: 12.6±1.3. Time effect: p = 0.515	N/A	Re-operation: 3/9 (33.3%) Reason for re-operation: 1 for symptomatic hypertrophy at 9 months, 2 for pain and cartilage thinning at 3 years
Barié et al., 2020 [41]	ACI-P	Pre-operative: 48.8 ± 9.6, 12 months: 82 ± 14.1, 24 months: 82.1 ± 18.1, last follow-up: 81.6 ± 12.2. Time effect: p = 0.018<br><br>Group effect: 3 months: p = 0.396, 12 months: p = 0.426, 24 months: p = 0.315, last follow-up: p = 0.204	N/A	N/A	Pre-operative: 57.3±11.9, 12 months: 92±7.2, 24 months: 87±19.1, last follow-up: 82±17.2. Time effect: p = 0.063<br><br>Group effect: 3 months: p = 0.265, 12 months: p = 0.185, 24 months: p = 0.137, last-follow-up: p = 0.289	Pre-operative: 3.2 ± 1.8, 12 months: 5.1 ± 2.1, 24 months: 5.6 ± 1.9, last follow-up: 5.6 ± 2. Time effect: p = 0.017<br><br>Group effect: pre-operative: p = 0.119, 3 months: p = 0.450, 12 months: p = 0.255, last follow-up: p = 0.455	Pre-operative: 30.3±1.2, 12 months: 31.9±1, 24 months: 32±1.1, last follow-up: 32± 0.7. Time effect: p = 0.018<br><br>Group effect: 3 months: p = 0.081, 12 months: p = 0.340, 24 months: p = 0.560, last follow-up: p = 0.368	Pre-operative: 11.9±0.6, 12 months: 11.9±0.4, 24 months: 11.7±0.5, last follow-up: 13.6±1. Time effect: p = 0.018<br><br>Group effect: 3 months: p = 0.315, 12 months: p = ≥.999, 24 months: p = 0.832, last follow-up: p = 0.186	N/A	Graft hypertrophy: 1/7 (14.3%), 2 Re-operation: 2/7 (28.6%), Reason for re-operation: synovectomy at 2 years and 7 years after ACI

Mean values are used unless specified. Abbreviations: SE, standard error; n, number of participants; IQR, inter-quartile range.
